# Marriage by Doctor's Consent

**Published:** 1896-05-09

**Authors:** 


### The Hospital

An Institutional Journal of

The Medical Sciences and Hospital Administration, with

AN EXTRA NURSING SUPPLEMENT.

Vol. XX.—No. 502.] [May 9, 1896.

### Marriage by Doctor's Consent.

In a presidential address delivered to the members of the Life Assurance Medical Officers' Association the other day, Dr. Douglas Powell proposed to apply the doctrine of "doctor's consent and approval" to a very personal and delicate set of social affairs. "In the course of our professional work," said Dr. Powell, "we are often consulted, either by individuals themselves or by those interested in them, whether they are in a fit state to marry; and a family practitioner is often in some perplexity as to how to advise in this important matter." The word "perplexity," perhaps, hardly does full justice to the situation in most cases of this kind. As a matter of fact, between the parents on the one hand, and the irresponsible and absolutely unscientific young people on the other, the unfortunate doctor is not merely "perplexed," but is not seldom entirely at his "wits' end." If he advises to please the young people, the parents may scowl upon and repudiate him; if he advises to please the parents, the young people will hardly be content without his "head on a charger"; while if he takes the line of high science and future possibility, both parents and young people will alike regard him as an unpractical adviser and pompous professor of scientific pedantry. What is to be done in a case of this kind? Dr. Douglas Powell is quite equal to the emergency. Life assurance, more especially that part of it which is medical, is the way out of the difficulty. Both parents and young people are willing that due provision should be made for emergencies when so serious a business as civilised marriage is in contemplation. In a doubtful case let the young man—and why not the young woman also?—be examined for life assurance. Then the family doctor, and even the occasional consultant, are both saved from a perplexing situation, and an absolutely disinterested and competent third party is called upon to solve the knotty question. If a man or a woman be not in a condition of health to emerge from the assurance specialist's consulting-room with a certificate of average healthiness—in other words, if the assurance doctor cannot pass the examinee as a "first-class life"—then it is at once made clear that he or she, at any rate at the moment of examination, is in no condition to enter upon the marriage state. It is not proposed that an unfavourable result of a first examination shall be a permanent bar to marriage. In such a case let the young man or the young woman submit to a probationary period of health improvement, not of course at the hands of the assurance medical officer; and if the probationary period result in the desired improvement, let the marriage take place, but if otherwise then let it be abandoned.

There is only one course more logical than the foregoing, and that Dr. Douglas Powell does not fail to point out. Instead of waiting until two young people have been "engaged" for a more or less lengthy period, and until the time of the marriage draws near, let the physical examination of both of them by an assurance medical officer precede the preliminary "engagement." "Of course," says Dr. Powell—and we do not detect any evidences of a sympathetic tear on the pages of his pamphlet—"of course, in such questions considerations of sentiment will come in," as indeed they will; "but," he adds, "with sentiment we have here nothing to do." It is but too true; and because none, not even the most brilliant of modern physiologists, have succeeded in reconciling science with sentiment in these matters, therefore it is that we are at such a tremendous discount in all manner of daily affairs, and are regarded too often as scientific nuisances, whose great function it is to act the part of "kill joys," and to make life "not worth the living."

But although we cannot get the dicta of science carried out in the absolute way in which they commend themselves to such scientific hierarchs as Dr. Douglas Powell, it does not therefore follow that science will continue to be altogether unheeded in this matter. Dr. Douglas Powell's "principles of right marriage," as they may be called, are at present to the multitude nothing more than mere "counsels of perfection." But there are always a few among civilised people who will strive to base their conduct even on "counsels of perfection." Then, as the application of these counsels in practice is seen to justify itself by results, other persons become convinced by facts, and so knowledge is extended, and a scientific principle converts itself into a commonplace of practice. That marriage among civilised people ought to be something more than a mere community of sentiment, that such a union ought not to be entered upon without adequate thought and preparation, not only for the future of the married persons themselves, but also for their probable offspring, surely goes without saying. Men and women are reasoning creatures; it is not for them to mate like the lower animals, without prevision and without preparation. All classes will do well to accept Dr. Douglas Powell's "Principles of Right Marriage" as altogether sound and wise in theory, and as being worthy of the fullest adoption in practice which the present state of civilisation will allow.

